# GPR56 Functions Together with α3β1 Integrin in Regulating Cerebral Cortical Development

**DOI:** 10.1371/journal.pone.0068781

**Published:** 2013-07-09

**Authors:** Sung-Jin Jeong, Rong Luo, Kathleen Singer, Stefanie Giera, Jordan Kreidberg, Daiji Kiyozumi, Chisei Shimono, Kiyotoshi Sekiguchi, Xianhua Piao

**Affiliations:** 1 Division of Newborn Medicine, Department of Medicine, Boston Children’s Hospital and Harvard Medical School, Boston, Massachusetts, United States of America; 2 Convergence Brain research Department, Korea Brain Research Institute (KBRI), Daegu, South Korea; 3 Department of Medicine, Boston Children’s Hospital and Harvard Medical School, Boston, Massachusetts, United States of America; 4 The Laboratory of Extracellular Matrix Biochemistry, Institute for Protein Research, Osaka University, Suita, Japan; National Center for Scientific Research Demokritos, Greece

## Abstract

Loss of function mutations in *GPR56*, which encodes a G protein-coupled receptor, cause a specific human brain malformation called bilateral frontoparietal polymicrogyria (BFPP). Studies from BFPP postmortem brain tissue and *Gpr56* knockout mice have previously showed that *GPR56* deletion leads to breaches in the pial basement membrane (BM) and neuronal ectopias during cerebral cortical development. Since α3β1 integrin also plays a role in pial BM assembly and maintenance, we evaluated whether it functions together with GPR56 in regulating the same developmental process. We reveal that loss of α3 integrin enhances the cortical phenotype associated with *Gpr56* deletion, and that neuronal overmigration through a breached pial BM occurs earlier in double knockout than in *Gpr56* single knockout mice. These observations provide compelling evidence of the synergism of GPR56 and α3β1 integrin in regulating the development of cerebral cortex.

## Introduction

The interaction between cells and their environment is essential to brain development. Dystroglycan and intergrins are two major cell surface receptors that mediate cell-extracellular matrix (ECM) interactions [1]–[4]. Recently, the family of adhesion G protein-coupled receptors (GPCRs) was identified as the third major category of ECM receptors [5], [6]. Adhesion GPCRs are characterized by the presence of a large extracellular region and a G protein proteolytic site (GPS) domain that cleaves the receptor into N- and C-terminal fragments [7]–[9]. Mutations in one such adhesion GPCR, GPR56, cause a specific human brain malformation called bilateral frontoparietal polymicrogyria (BFPP) [10]. BFPP is a recessively inherited genetic disorder affecting human cerebral cortical development and characterized by disorganized cortical lamination that is most severe in the frontal and parietal lobes [10]–[13]. Histological analyses of postmortem human BFPP brain samples and *Gpr56* knockout mice indicated that the histopathology of BFPP is a cobblestone-like brain malformation [14], [15].

Cobblestone lissencephaly, also called type II lissencephaly, is defined as aberrant migration of cortical neurons through breaches in the pial basement membrane (BM), resulting in neuronal ectopias on the surface of the brain [16]. Cobblestone cortex is typically seen in three distinct human congenital muscular dystrophy syndromes: Muscle-eye-brain disease (MEB), Fukuyama-type muscular dystrophy (FCMD), and Walker-Warburg syndrome (WWS) [16]. These three disorders are autosomal recessive diseases that encompass congenital muscular dystrophy, ocular malformations, and cobblestone lissencephaly. MEB, FCMD, and some WWS cases are caused by aberrant glycosylation of α-dystroglycan, a receptor for laminin [17]–[20].

Defects in some members of the integrin family and their ligand, laminin, have been implicated in the pathogenesis of BM breakdown and neuronal ectopias [21]–[24]. Furthermore, our previous work demonstrated that loss of GPR56 diminishes the adhesion of rostrally derived granule cells to laminin [25]. Integrin α3 (gene symbol, *Igta3*) always associates with β1 integrin to form the α3β1 dimeric protein on the cell surface, serving as one of the major receptors for laminin [26], [27]. We therefore investigated how GPR56 functionally interacts with α3β1 integrin *in vivo* by studying *Gpr56* and *Itga3* compound mutant mice. We demonstrate that loss of α3β1 integrin exacerbates the *Gpr56*-associated cortical phenotype in a dose dependent manner, indicating that the two receptors function synergistically during cortical development.

## Results

### Loss of *Itga3* Enhances the Cortical Phenotype Associated with *Gpr56* Deletion

**Figure 1:**
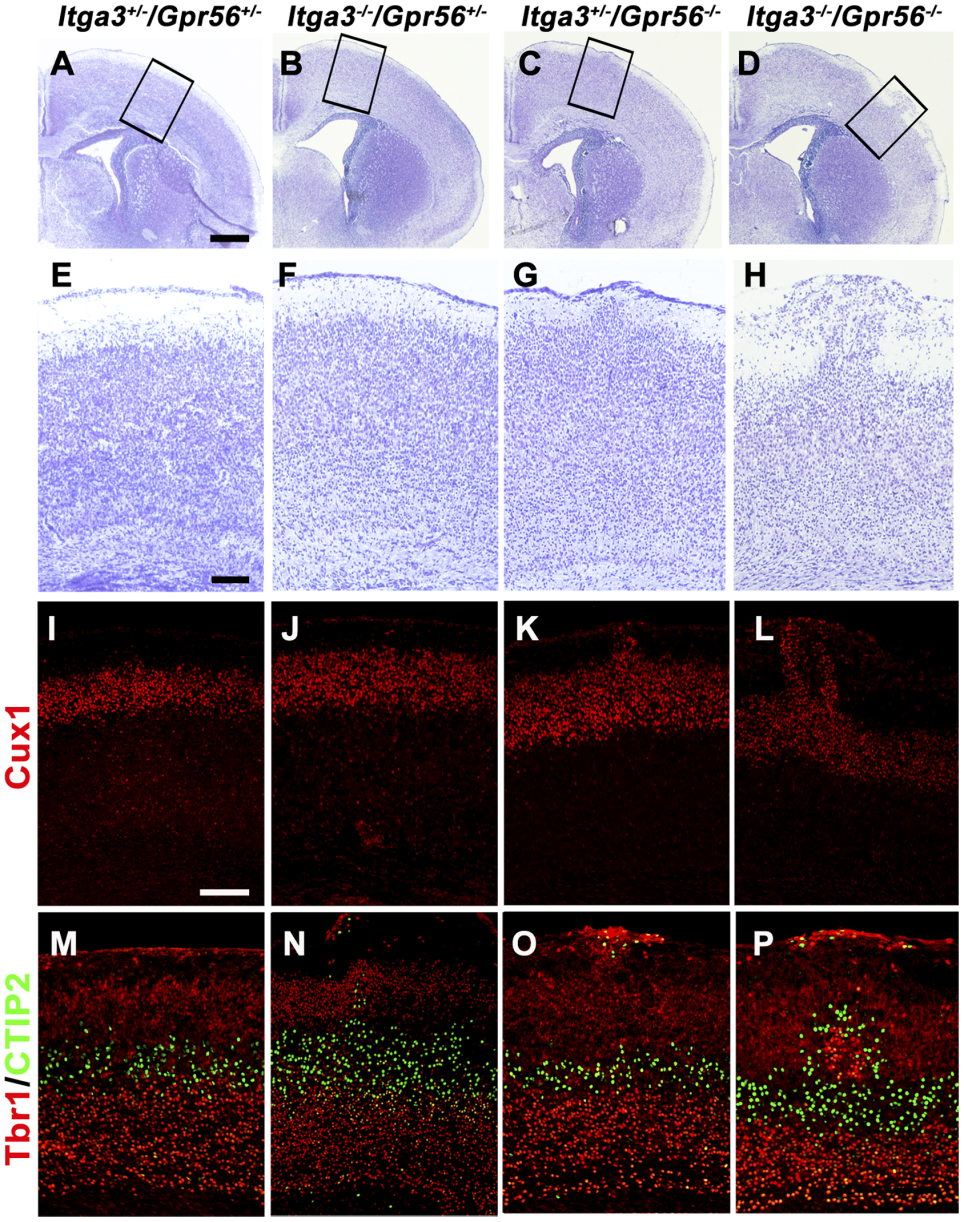
Dual deletion of *Itga3* and *Gpr56* results in cortical lamination defect. (A–D) Nissl staining on coronal sections of P0 *Itga3^+/−/^Gpr56^+/−^* (A), *Itga3^−/−/^Gpr56^+/−^* (B), *Itga3^+/−/^Gpr56^−/−^* (C), and *Itga3^−/−/^Gpr56^−/−^* (D). Normal cortical lamination was observed in *Itga3^+/−/^Gpr56^+/−^* (A) and *Itga3^−/−/^Gpr56^+/−^* (B), while cortical ectopias were seen in *Itga3^+/−/^Gpr56^−/−^* (C) and *Itga3^−/−/^Gpr56^−/−^* (D) brains. (E–H) Higher magnification of boxed regions in A–D. (I–L) Cux1(red) IHC. (M–P) Double IHC of Tbr1 (red) and CTIP2 (green). In contrast to the well organized cortical layers in *Itga3^+/−/^Gpr56^+/−^* brains (I and M), regional overmigration of both superficial and deeper layer neurons were observed in *Itga3^−/−/^Gpr56^+/−^* (J and N), *Itga3^+/−/^Gpr56^−/−^* (K and O), and *Itga3^−/−/^Gpr56^−/−^* (L and P) brains. Scale bars: A–D, 500 µm; E–P, 100 µm.

Since *Itga3* ^−/−^ mice die at birth with kidney and lung defects and moderate skin blistering, we crossed *Gpr56* ^−/−^ with *Itga3* ^+/−^ mice and intercrossed the F1 offspring to generate compound mutant mice [28]. Genotype for various mutant mice was confirmed by PCR as previously described [14], [28]. The absence of protein expression was verified by immunohistochemistry (IHC) for α3 integrin (Figure S1) and western blot analysis for GPR56 (Figure S2). First, we evaluated the overall cortical lamination at P0 by Nissl staining of various coronal sections of compound mutant mouse brains. Consistent with our previous study, *Gpr56* single knockout mice revealed neuronal ectopias (Table 1), while *Gpr56* heterozygous mice appear phenotypically normal (data not shown) [14]. Loss of α3 integrin was previously shown to result in poor neuronal migration and the formation of heterotopia [29], [30]. However, we did not observe any discernible lamination defect in *Itga3* single knockout mouse brains, except mild neuronal overmigration in one out of 11 P0 *Itga3* ^−/−/^ *Gpr56* ^+/−^ brains (Figure 1N and Table 1). Double heterozygous mice (*Itga3* ^+/−/^ *Gpr56* ^+/−^) were also lacking any obvious brain phenotype (Table 1).

**Table 1:** Penetrance of cortical dysplasia in *Itga3/Gpr56* compound mutant mice.

Number of brains with ectopia/Number of total brains analyzed						
Stage	*Itga3^+/−/^Gpr56^+/−^*	*Itga3^−/−/^Gpr56^+/+^*	*Itga3^−/−/^Gpr56^+/−^*	*Itga3^+/+^/Gpr56^−/−^*	*Itga3^+/−/^Gpr56^−/−^*	*Itga3^−/−/^Gpr56^−/−^*
**E10.5** *					0/3	0/3
**E11.5**				0/4	4/4	4/4
**E12.5**				0/4	10/10	5/5
**E12.8** **				4/4		
**E13.5**				19/19	6/6	3/3
**E14.5**				22/22	8/8	5/5
**E16.5**	0/26	0/5	0/11	26/26	38/38	17/17
**P0**	0/23	0/5	1/11	32/32	30/30	26/26
**Summary**	**0/49**	**0/10**	**1/22**	**103/111**	**96/99**	**60/63**

* 10am on the 10^th^ day of vaginal plugging is assigned as embryonic day (E) 10.5;

** 6pm on the 12^th^ day of vaginal plugging is assigned as E 12.8.

Interestingly, *Itga3^+/−/^Gpr56^−/−^* and *Itga3^−/−/^Gpr56^−/−^* mice showed more severe cortical defects than what is observed in *Gpr56* single knockout mouse brains (Figure 1C, D, G and H). To further reveal the cortical lamination defects, we performed layer marker staining with Cux1 for layer II–IV, CTIP2 for layer V, and Tbr1 for layer II–III and layer VI neurons [31]–[33]. Cux1-, Tbr1-, as well as CTIP2-positive neurons were detected in the ectopic clusters, indicating that the developmental abnormalities affects to both superficial and deeper layer neurons (Figure 1J–L and N–P).

**Figure 2:**
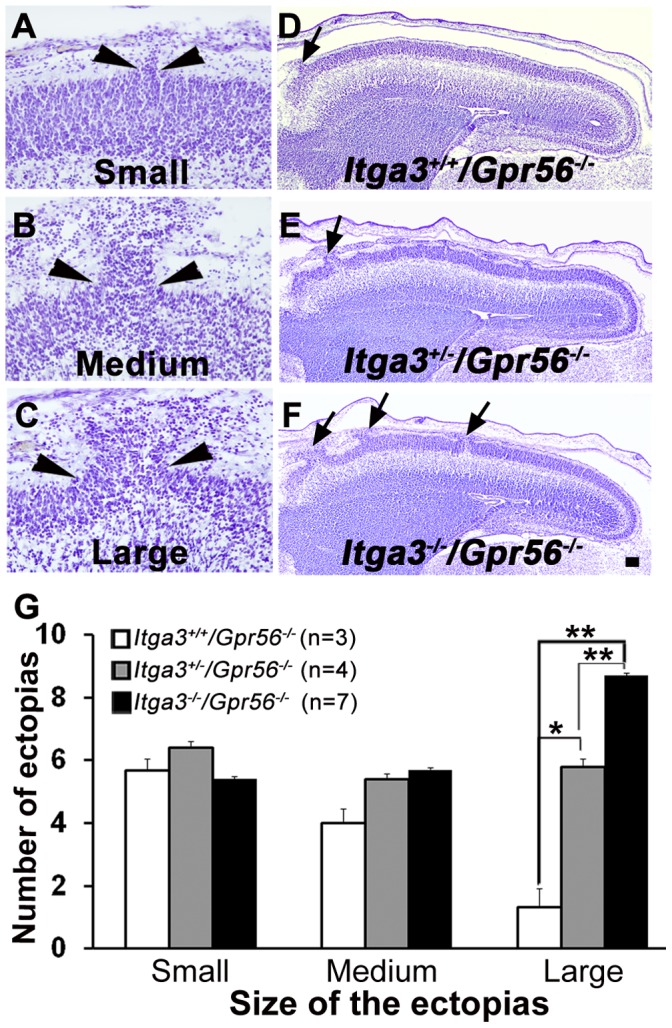
More severe dysplasias were observed in *Itga3*/*Gpr56* compound mutant mice. (A–C) Representatives of small (A), medium (B), and large (C) ectopias. The base of the neuronal ectopia between the two arrowheads was measured and grouped into small (<50 µm), medium (50–100 µm), and large (>100 µm) categories for semi-quantification. (D–F) Nissl staining on sagittal sections of E16.5 *Itga3^+/+^/Gpr56^−/−^* (D), *Itga3^+/−/^Gpr56^−/−^* (E) and *Itga3^−/−/^Gpr56^−/−^* (F) brains. Scale bar, 100 µm. (G) Semi-quantification data are presented as means ± S.E.M. *P = 0.17, **P<0.05, t-test. Cortical dysplasias were more severe in *Itga3^+/−/^Gpr56^−/−^* and *Itga3^−/−/^Gpr56^−/−^* than *Gpr56^−/−^* single knockout mice. N indicates number of embryos examined.

To further quantify the severity of the cortical defects in various compound mutant mice, we performed semi-quantitative analyses of Nissl-stained brain sections at E16.5. We defined the cortical ectopias as small, medium, and large based on the width of the ectopic outgrowth (Figure 2A–C). Compared to *Gpr56* single knockout mice, there was a significantly greater number of large size cortical ectopias in *Itga3* ^+/−/^ *Gpr56* ^−/−^ and *Itga3* ^−/−/^ *Gpr56* ^−/−^ mice, with *Itga3* ^−/−/^ *Gpr56* ^−/−^ mice being the most severely affected (Figure 2D–G). Again, we did not identify any discernible cortical phenotype in *Itga3* ^−/−/^ *Gpr56* ^+/+^, *Itga3* ^−/−/^ *Gpr56* ^+/−^, and *Itga3* ^+/−/^ *Gpr56* ^+/−^ mice at E16.5 (Table 1). These data confirmed that cortical ectopias are only associated with *Gpr56* mutation and that loss of α3β1 integrin enhances the *Gpr56*-associated cortical phenotype, with each additional allele loss corresponding to a more severe phenotype, suggesting a possible functional interaction between α3β1 integrin and GPR56 during cortical development.

### Earlier Pial BM Breakdown with Associated Neuronal Overmigration was Observed in the *Itga3^+/−/^Gpr56^−/−^* and *Itga3* ^−/−/^ *Gpr56* ^−/−^ Neocortices

**Figure 3:**
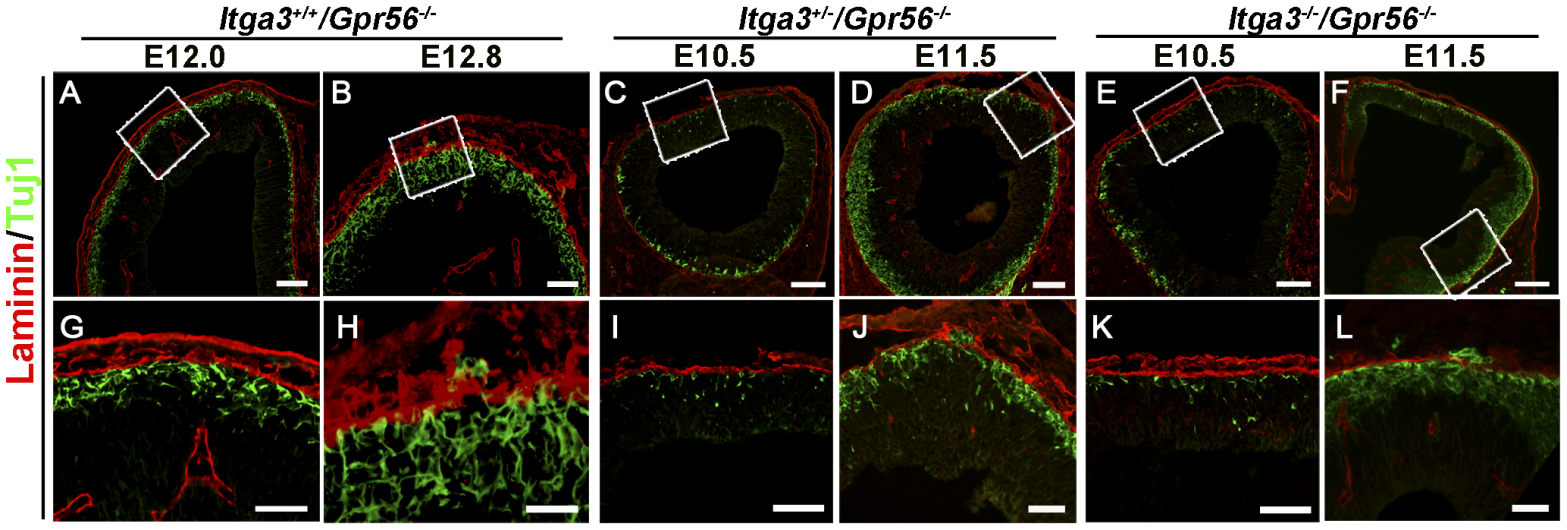
Earlier pial BM breakdown with concurrent neuronal overmigration in double mutant mouse neocortices. (A–F) Double IHC of Tuj1 (green) and laminin (red) during E10.5–E12.8 coronal sections of various compound mutant mouse brains as indicated in the figure. Neuronal overmigration and pial BM breaches were first observed at E12.8 in *Gpr56* single knockout mice (B), whereas they occur at E11.5 in both *Itga3^+/−/^Gpr56^−/−^* and *Itga3^−/−/^Gpr56^−/−^* mice (D and F). (G-L) Higher magnification of boxed regions in A–F. Scale bars: A–F,100 µm; G and H, 25 µm; I-L, 50 µm.

We have previously showed that the pial BM was properly formed in *Gpr56* single knockout mouse embryonic brains before E12.5 (10 am on the 12^th^ day of vaginal plugging), and regional pial BM breaches started to occur at E12.8 (6 pm on the 12^th^ day of vaginal plugging) [14]. Based on the fact that more severe cortical ectopias were observed in *Itga3* ^+/−/^ *Gpr56* ^−/−^ and *Itga3* ^−/−/^ *Gpr56* ^+/−^ mice, we hypothesized that pial BM breaches would occur earlier in these two mutant mice. To test this hypothesis, we performed a detailed time course study of the occurrence of the breached pial BM and overmigrated neurons in double mutant mice. Double IHC of Tuj1 and laminin revealed that the pial BM was well formed in E10.5 neocortices of double mutant mice (Table 1, Figure 3C, E, I and K). Regional breakdown of the pial BM with concurrent neuronal overmigration was observed at E11.5 (Figure 3D, F, J, and L). Again, we observed migrating neurons piercing through a well formed pial BM at approximately E12.8 in *Gpr56* single knockout mice (Figure 3B and H) [14]. The notably earlier onset of BM breaching and neuronal overmigration seen in *Gpr56* and *Itga3* double mutant mice further supports the notion that α3 integrin has a cooperative function with GPR56 during cortical development. Although the onset of BM breaching and neuronal overmigration was observed at a similar developmental stage by immunostaining in both *Itga3^+/−/^Gpr56^−/−^* and *Itga3* ^−/−/^ *Gpr56* ^−/−^ mice, the number of larger ectopias was significantly higher in *Itga3* ^−/−/^ *Gpr56* ^−/−^ mice (Figure 2D–G). The possible explanation could be that immunostaining is not sensitive enough to detect the difference in the onset of pial BM breakdown between both *Itga3^+/−/^Gpr56^−/−^* and *Itga3* ^−/−/^ *Gpr56* ^−/−^ mice.

**Figure 4:**
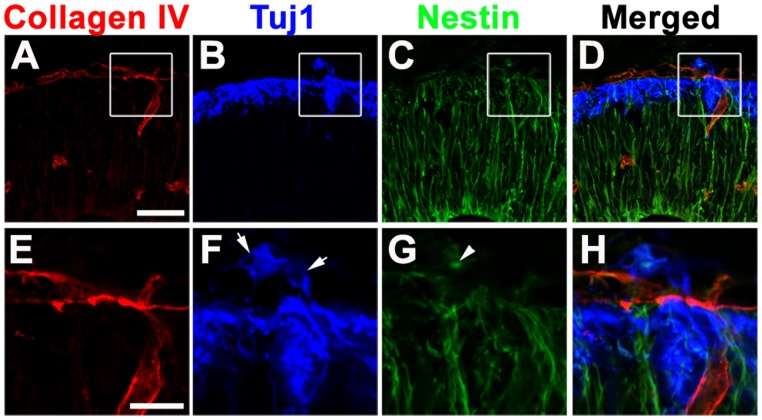
Concurrent events of neuronal overmigration and radial glial endfeet misplacement. (A–D) Triple IHC of collagen IV (red, A), Tuj1(blue, B), and Nestin (green, C) on E11.5 *Itga3^−/−/^Gpr56^−/−^* neocortices. A cluster of neurons and radial glial endfeet were detected beyond the appeared to be an intact pial BM. (E–H) Higher magnification of boxed regions in A–D. Scale bars : A–D, 50 µm; E–H, 25 µm.

To investigate the relationship of migrating neurons, radial glial endfeet, and pial BM, we performed triple IHC on E11.5 *Itga3^−/−/^Gpr56^−/−^* neocortices. Interestingly, both migrating neurons and radial glial endfeet were protruded through seemingly well formed pial BM (Figure 4D and H). Taken together, our data suggests a possibility that migrating neurons and radial glial endfeet pierce through a previously well formed pial BM.

### Loss of *Itga3* Disrupted Collagen III-mediated Neuronal Migration Inhibition

**Figure 5:**
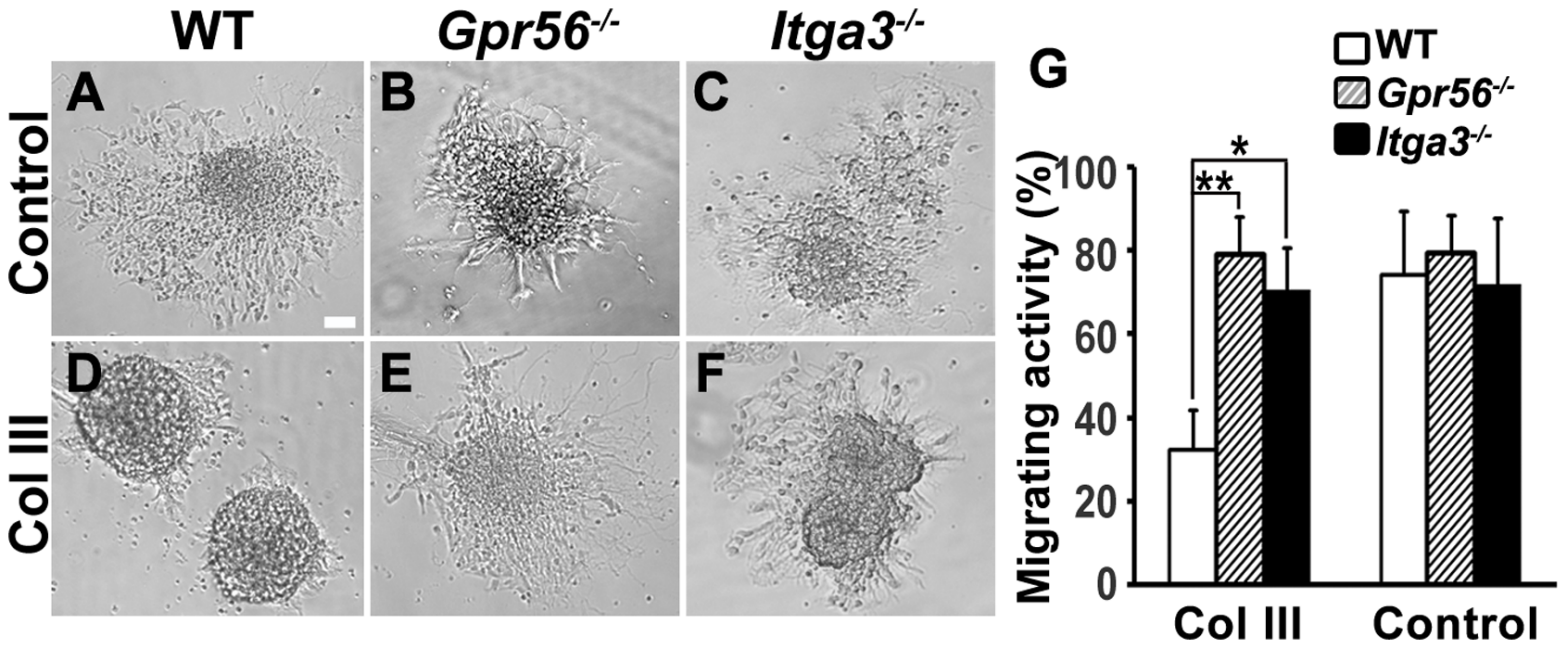
Loss of *Itga3* attenuates Collagen III-mediated neuronal migration inhibition. (A–F) Neurosphere migration assays were performed with neural progenitor cells (NPC) derived from WT (A and D), *GPR56^−/−^*(B and E), and *Itga3^−/−^* (C and F) mouse cortices in the presence of collagen III (D–F) or carrier solution (A–C). Representative images are shown. (G) The degree of collagen III-mediated migration inhibition was quantified as a percentage of the migrating neurospheres. Data are presented as mean ± S.D.; n = 3. *P = 0.04, **P = 0.002, Student t-test.

On a cellular level, the binding of GPR56 and its ligand collagen III causes an inhibition of neuronal migration [34]. To elucidate the synergistic function of GPR56 and α3β1 integrin at the cellular level, we questioned whether loss of α3 integrin affects collagen III-mediated neuronal migration inhibition. Neuronal migration assays were conducted to determine whether deleting *Itga3* would lead to a decrease in the inhibition of migration. Neurospheres established from E13.5 cortices of either wild type, *Gpr56* ^−/−^, or *Itga3* ^−/−^ mice were cultured in neuron culture medium containing 84 nM of purified collagen III or control solution (acetic acid). After two days in culture, the neurospheres were assessed for being either positive or negative for migration, using criteria described previously [34]. Neurospheres derived from *Itga3^−/−^* cortices had a significantly diminished migration inhibition in comparison to wild type (Figure 5E, F, and G).

### Integrin α3β1 does not Bind Directly to Collagen III

**Figure 6:**
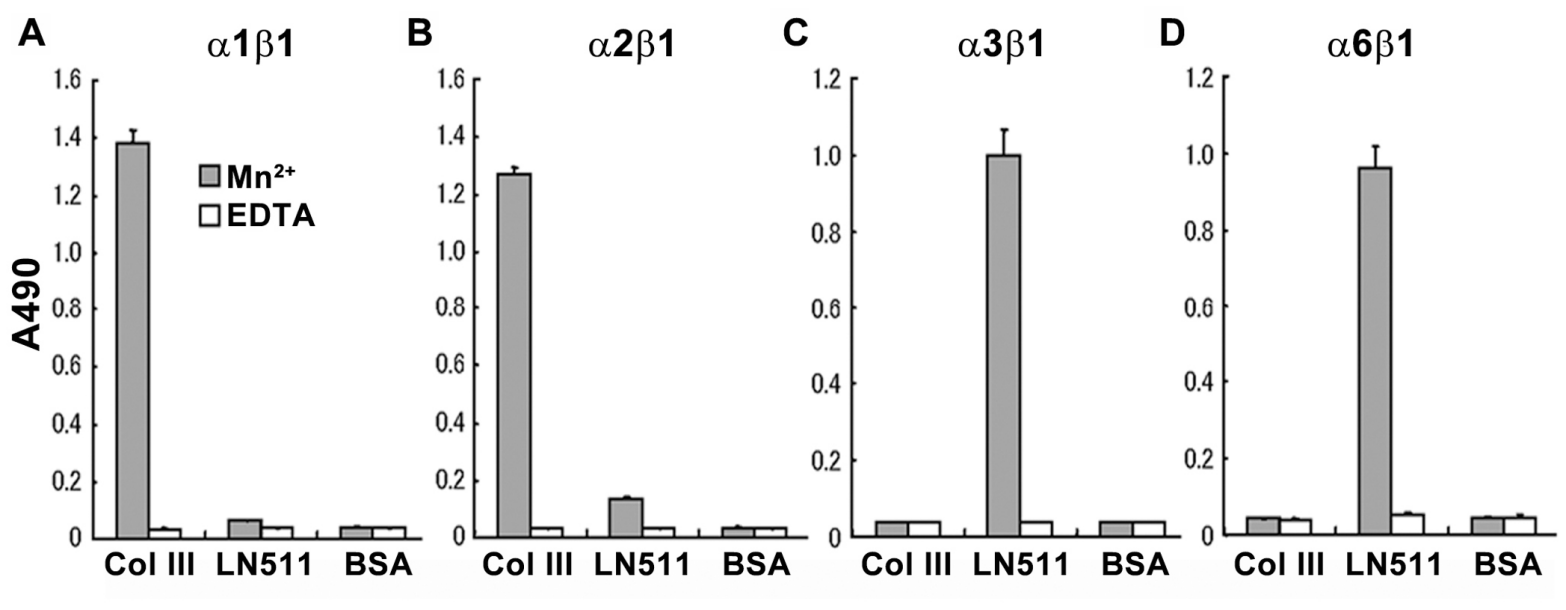
The binding of integrins to collagen III and laminin-511. (A–D) Integrins α1β1 and α2β1 bind to collagen III (A, B). In contrast, integrins α3β1 and α6β1 bound to laminin-511 (C, D). Both collagen III and laminin binding only occur in the presence of 1 mM Mn^2+^. The results are shown as mean ± S.D.; n = 3.

Thus far, we have demonstrated that GPR56 functions together with α3β1 integrin in regulating cerebral cortical development. Mechanistically, there are two possibilities to account for this synergistic activity of the two receptors: (1) GPR56 and α3β1 integrin function in the same receptor complex and bind collagen III as a common ligand; (2) the two receptors act indirectly through an unknown mediator, which requires the activation of GPR56 pathway. To investigate the first possibility, we conducted a solid phase binding assay using recombinant α1β1 and α2β1 integrins, the natural receptors for collagen III, as the positive control [35], [36]. Recombinant human α1β1, α2β1, α3β1, and α6β1 integrins were tested for their ability to bind to human collagen III and recombinant human laminin-511 coated on 96-well plates in the presence of 1 mM Mn^2+^ or 10 mM EDTA. Integrins α1β1 and α2β1 bound to collagen III as expected (Figure 6A and B). However, α3β1 and α6β1 integrins did not bind to collagen III (Figure 6C and D). The biological activities of α3β1 and α6β1 integrins were confirmed by their binding to laminin-511 (Figure 6C and D). This result is consistent with our previous publication, in which loss of GPR56 reduced the adherence of granule cells to integrin ligands, laminin and fibronectin, without direct binding to these two substrates (further discussed below) [25].

To determine the nature of the cooperation between these proteins, we next investigated the localization of the two proteins in the developing neocortex. We performed double IHC of GPR56 and α3 integrin on E10.5 and E11.5 mouse brain sections. The specificity of anti-α3 integrin was confirmed on *Itga3* ^−/−^ mouse embryonic brains as well as 1 day in vitro (1DIV) cultured progenitor cells (Figure S1). Similar to the expression pattern of GPR56, α3 integrin was present throughout the cerebral wall at both stages, with strong signals in radial glial cells as well as on the basal surface of the neocortex where preplate neurons reside (Figure S3) [37]. GPR56 and α3 integrin were highly colocalized in radial glial cells and rostral preplate neurons (Figure S3). To further demonstrate that GPR56 and α3 integrin were co-expressed in the same cells, we performed a double immunostaining using GPR56 and α3 integrin antibodies on 1DIV cultured progenitor cells. As shown in Figure S3U, some α3 integrin-positive cells indeed express GPR56.

**Figure 7:**
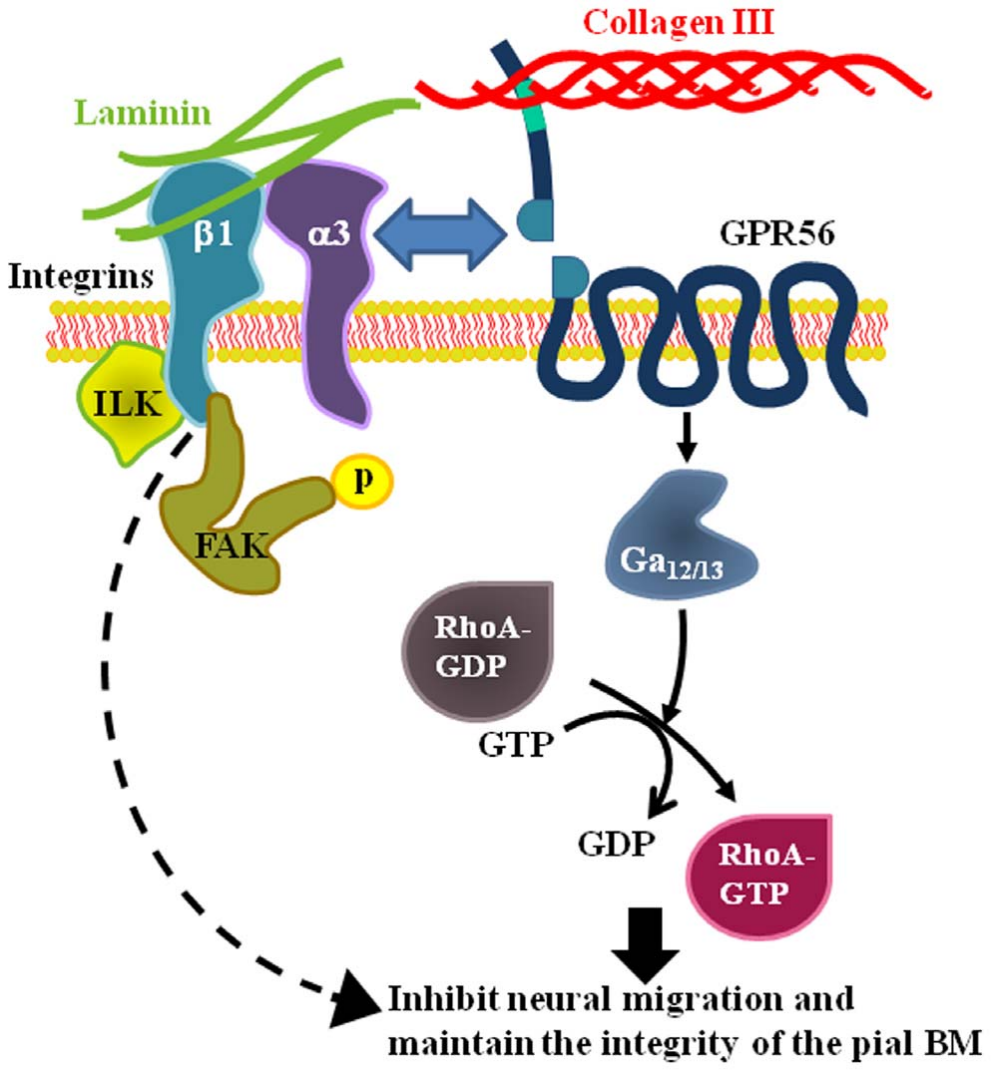
Synergic activity of GPR56 and α3β1 integrin during cortical development. The regulation of neural migration and the maintenance of the pial BM integrity are accomplished through the coordinated activities of both GPR56 and α3β1 integrin pathways. The binding of GPR56 to collagen III activates RhoA via Gα_12/13_ pathway, thus inhibits neural migration. Upon the binding to laminin, α3β1 integrin induces FAK phosphorylation.

Taken together, our data suggests that α3β1 integrin functions together with GPR56 in regulating cortical development, probably via an unknown mediator that requires the activation of GPR56 pathway (Figure 7).

## Discussion

This study demonstrates synergistic activities of GPR56 and α3β1 integrin during cerebral cortical development. A more severe cortical phenotype was uncovered in compound mutants reflecting the fact that several receptors must function together in regulating cortical development. The requirement of both α3β1 integrin and GPR56 for the proper cortical development illustrates that the regulation of the pial BM integrity and cortical lamination are integrated processes and suggests a link with two different signaling pathways.

Integrins are heterodimeric transmembrane receptors composed of noncovalently associated α and β chains [38]. While some α chains bind to multiple β chains, α3 integrin is always associated with β1 to form a mature α3β1 integrin receptor on the cell surface [39]. Integrin α3β1 was described as a promiscuous receptor for many ligands, among which is laminin-5 [40]. However, α3β1 integrin does not bind directly to collagen III, which serves as the ligand of GPR56 (Figure 6). This is consistent with our previous study on the developing cerebellum [25]. GPR56 is expressed specifically in the rostral granule cells perinatally and absence of GPR56 causes loss of adhesion of rostrally derived granule cells to laminin-1 and fibronectin, known to be the ligands of integrin. However, this cell-matrix adhesion is not through direct binding of GPR56 to the matrix proteins because neither addition of soluble GPR56 to the media in the granule cell adhesion assay nor overexpression of full-length mouse GPR56 in HEK 293T cells altered cell adhesion to laminin-1 and fibronectin [25]. Taken together, our previous and current study results suggest a functional link between integrin and GPR56, despite the lacking of a common binding partner.

Loss of α3 integrin was previously shown to result in poor neuronal migration and the formation of heterotopia [29], [30]. We did not observe any obvious cortical defect in *Itga3* single knockout mice, presumably due to differences in mouse genetic background (129/BL6 vs 129/BL6/FvB). However, *Gpr56* and *Itga3* double knockout mice developed a more severe cortical malformation manifested by neuronal ectopias and an earlier occurrence of pial BM breachment and neuronal overmigration, compared to *Gpr56* single knockout mice. Our finding is consistent with other reports relevant to α3 integrin. For example, α3 and α6 integrins double knockout mice exhibit regional breakdown of pial BM with concurrent neuronal ectopias [22]. Studies of β1 integrin, as well as integrin associated downstream partners, integrin-linked kinase (ILK) and focal adhesion kinase (FAK), also support the notion that α3β1 integrin is essential to maintaining and reforming the pial basement membrane, with ILK, FAK, and β1 integrin knockout mice exhibiting neuronal ectopias on the brain surface with concurrent regional fragmentation in the basal lamina, features of cobblestone-like cortical malformation [41]–[43].

The leading pathology of cobblestone-like cortical malformation is thought to be a defective pial BM [16]. However, recent literature suggests that abnormal neuronal migration could be partly responsible [44], [45]. We have previously showed that (1) a gradient expression of GPR56 in preplate neurons that matches the regional cortical defects associated with loss of GPR56, in spite of the fact that no such pattern is apparent in the radial glia [37]; (2) neurons have direct contact with the pial BM during early cortical development [34]; and (3) the interaction of GPR56 and collagen III inhibits neuronal migration [34]. In this study, we further demonstrated the expression of α3 integrin in the preplate neurons as well as an attenuated collagen III-mediated neuronal migration inhibition in *Itga3* ^−/−^ neural progenitor cells. Taken together, it is likely that GPR56 functions together with α3β1 integrin in mediating the interaction between the pial BM and preplate neurons as well as radial glial endfeet, thus defining the boundary between the neocortex and the meninges while providing a framework for the developing cortex (Figure 7).

## Materials and Methods

### Ethics Statement

Experiments were performed in accordance with National Institutes of Health guidelines for the care and use of laboratory animals, and with approval of the Animal Care and Use Committee of Boston Hospital Boston (approval ID: A3303–01).

### Mice

*Gpr56* knockout mice were obtained from Genentech, maintained in a mixed genetic background of 129/BL6/FvB. *Itga3* knockout mice were generated on a 129 background [28]. Crossing *Gpr56* ^−/−^ with *Itga3* ^+/−^ produced *Gpr56/Itga3* double mutant mice in a mixed 129/BL6/FvB genetic background. Fetal stage was calculated from the day when a vaginal plug was observed (considered as E0.5).

### Antibodies

Mouse anti-GPR56 (H11, 1∶200) [37], rabbit anti-alpha 3 integrin (EMD Millipore Co., 1∶700. It is worth noting that there were significant variations between lots of Millipore rabbit anti-α3 integrin antibody, in which only those that worked for western blots also worked for IHC.), rabbit anti-cux1 (Santa Cruz Biotechnology, 1∶50), rat anti-CTIP2 (Abcam, 1∶500), rabbit anti-laminin (Sigma, 1∶250), rabbit anti-Tbr1 (gift from R. Hevner, University of Washington, Seattle, WA, USA, 1∶500), mouse or rabbit anti-Tuj1 (Covance, 1∶1000), goat anti-Collagen IV (Southern Biotech, 1∶10), mouse anti-Nestin (BD Transduction Lab, 1∶200). A biotinylated anti-Velcro rabbit polyclonal antibody (against ACID/BASE coiled-coil peptides contained in recombinant integrins) was kindly provided by Dr. Junichi Takagi (Institute for Protein Research, Osaka University, Osaka, Japan) [46]. A streptavidin–HRP conjugate was purchased from ZYMED Laboratories.

### Histology and Immunohistochemistry

Histological analysis was carried out as previously described [14], [37]. Embryonic brains of E10.5 to E12.5 were fixed for frozen sectioning in 4% PFA for 2–3 hr at 4°C. E16.5 brains and P0.5 brains were fixed in 4% PFA at 4°C for 48 hrs or 24 hrs, respectively, cryoprotected by 30% sucrose in PBS at 4°C sinking, embedded in OCT compound (Tissue Tek, Sakura Finetek USA INC.), and stored at −80°C until sectioned. For double IHC of α3 integrin and GPR56 at E10.5 and E11.5, the sections were retrieved by boiling for 8 min followed by cooling them down at room temperature (RT) for 30 min. After washing the slides with PBS three times, they were incubated with 1% SDS for 5 min followed by washing three times again with PBS. The sections were incubated with rabbit anti-α3 integrin and mouse anti-GPR56 antibodies overnight at 4°C and washed with normal PBS one time, PBS containing 2.7% NaCl (instead of 0.8% NaCl for normal PBS) twice, and normal PBS once. Primary antibodies were visualized by goat anti-rabbit Alexa-546 and goat anti-mouse Alexa-488 secondary antibodies. Nuclei were stained with Hoechst 33342 (Invitrogen, 1∶2000). Images were captured using a Nikon 80i upright microscope or a Olympus confocal FluoView Laser System (FluoView FV1000).

For double IHC of Tbr1 and CTIP2, TSA-TMR (Perkin Elmer) was used to amplify the Tbr1 signal in a 1∶50 dilution followed by incubation of peroxidase-conjugated goat anti-rabbit IgG antibody (Sigma) for 30 minutes.

### Semi-quantitative Measurements of Cortical Dysplasia

Neuronal ectopias in various compound mutant brains at E16.5 were captured by a Nikon 80i upright microscope and subjected to blind quantitative analysis. Sagittal cryostat sections (8 µm) were obtained serially from lateral to medial, starting from the point when the posterior horn of the lateral ventricle could be clearly viewed. Every six continuous sections were collected and stained with 0.1% cresyl violet/0.5% acetic acid for semi-quantitative analysis. For each brain, 13 sections in total were analyzed. The base of the neuronal cluster invading the marginal zone was measured and the ectopic cluster was defined as large (>100 µm), medium (50–100 µm), and small (<50 µm) (Figure 2 A–C). Data are presented as means ± S.E.M. Statistical analysis was performed using t-test with *P*<0.05 considered significant.

### Vector Construction

Expression vectors for recombinant human α3β1 and α6β1 integrins were prepared as described previously [47]. The cDNAs encoding the extracellular domains of human integrin α1 and α2 subunits were generated by reverse transcription-PCR using pre-made double-stranded cDNAs derived from human fetal tissues (Clontech). The primers for human integrin α1 subunit were 5′-AAGGTACCACCATGGCCCCTCGGCCCCGCGCCCGCCCA-3′ and 5′- TTTGCGGCCGCGCCCGGTAGCCCATCTTTGGATATTTGA-3′. The primers for human integrin α2 subunit were 5′- AAGGTACCACCATGGGGCCAGAACGGACAGGGGCCGCGCCGCT-3′ and 5′-TTTGCGGCCGCGGCTTTCTCATCAGGTTTCATTATCAT-3′. The resulting cDNA fragments were inserted into the *Kpn*I/*Not*I sites of pcDNA3.1(+)-ACID-FLAG vector followed by sequence verification.

### Expression and Purification of Recombinant Proteins

The recombinant human integrins were designed as heterodimeric soluble proteins composed of the extracellular domains of integrins with ACID/BASE coiled-coil peptides and FLAG/6×His tag sequences at their C-termini for solid-phase binding assays [46], [47]. Recombinant integrins were produced using a FreeStyle™ 293 Expression System (Invitrogen). Briefly, 293F cells were simultaneously transfected with expression vectors for α and β subunits using 293fectin (Invitrogen), according to the manufacturer’s instructions, and grown in serum-free FreeStyle™ 293 Expression medium for 72 h. The conditioned media were collected and clarified by centrifugation, and then subjected to affinity chromatography using an anti-FLAG® M2-agarose (Sigma) column. The column was washed with Tris-buffered saline (TBS) containing 1 mM MgCl_2_ and 1 mM CaCl_2_, TBS (+). The bound proteins were eluted with TBS (+) containing 100 µg/ml FLAG peptide (Sigma) and dialyzed against TBS. Recombinant human laminin-511 was produced using a FreeStyle™ 293 Expression System (Invitrogen) and purified from conditioned media as described previously [48]. The protein concentrations of all the recombinant products were determined using a BCA protein assay kit (Thermo Scientific) using bovine serum albumin (BSA) as a standard. The expression vectors for recombinant human laminin-511 were constructed as described previously [48], [49].

### Solid-phase Binding Assays

Ninety six-well microtiter plates were coated with human type III collagen (BD Bioscience) and recombinant human laminin-511, and BSA (10 nM, 50 µl/well) overnight at 4°C, and then blocked with TBS containing 1% BSA and 0.02% Tween-20 for 1 h at RT. Next, the plates were washed with TBS containing 0.1% BSA, 0.02% Tween-20, and 1 mM MnCl_2_ (Buffer A) or TBS containing 0.1% BSA, 0.02% Tween-20, and 10 mM EDTA (Buffer B). Diluted integrins (10 nM, 50 µl/well) were added to the plates and allowed to bind to the substrate-adsorbed ligand proteins in the presence of 1 mM MnCl_2_ or 10 mM EDTA for 3 h at RT. The plates were washed three times with Buffer A or Buffer B, and the bound integrins were quantified by an enzyme-linked immunosorbent assay as reported previously [47]. Briefly, the wells were incubated with the biotinylated anti-Velcro antibody (1 µg/ml, 50 µl/well) for 30 min at RT in Buffer A, washed three times with Buffer A and incubated with HRP-conjugated streptavidin (0.33 µg/ml, 50 µl/well) for 15 min. After three washes with Buffer A, the bound antibodies were quantified by measuring the absorbance at 490 nm after incubation with *o*-phenylenediamine.

### Primary Neural Culture and Immunocytochemistry (ICC)

Primary neural culture was performed as previously described [37]. Briefly, cortical cells were harvested from E13.5 mouse cortices with the meninges removed. Dissociated cells were seeded on 10 ml tissue culture dish at 37°C for 10 min to deplete the fibroblasts. The cells (1×10^5^/ml) were placed on cover glasses pre-coated with poly-D-lysine (100 µg/ml) and cultured in neural culture medium (neurobasal medium supplemented with B27, 1% penicillin/streptomycin, and 1% L-glutamate) for one day (1DIV).

To perform ICC, the cultured primary neuronal cells were fixed in cold 95% ethanol and 5% glacial acetic acid for double ICC of H11 and α3 integrin, followed by three washes with PBS. Cells were permeabilized with 0.1% Triton-X 100 in PBS for 10 min followed by three washes with PBS. After blocking with 10% goat serum, 1% BSA, and 0.1% Triton-X100 in PBS for 30 min, the primary antibodies were incubated at 4°C overnight and visualized by appropriate fluorophore-conjugated secondary antibodies (Invitrogen, 1∶1000). Nuclei were stained with Hoechst 33342 (Invitrogen, 1∶2000). Images were captured using a Nikon 80i upright microscope.

### Migration Assay

Neurosphere generation and migration assay were performed as previously described [34]. Briefly, E13.5 mouse cerebral cortex was separated with the overlying meninges removed. Individual embryonic mouse cortex was cultured in neuron culture media (DMEM supplemented with B27, 1% penicillin/streptomycin, 1% L-glutamate, and 10% FBS). After genotyping, the mouse cortices were pooled blindly into two groups (wild type and *Itga3* knockout). The neurospheres were plated in a 48-well dish precoated with 100 µg/ml poly-D-lysine (PDL) and cultured in neuron culture medium overnight to allow the neurospheres adhere to the PDL substrate. The culture medium was then changed to the experimental mediums: neuron culture medium with 84 nM purified human collagen III (Abcam), or with carrier solution (acetic acid) as control. The neurospheres were imaged and the number of migrating neurospheres was quantified, as detailed previously.

## Supporting Information

Figure S1 **Integrin α3 immunostaining on** ***Itga3*** **^+/+^,** ***Itga3*** **^−/−^, and** ***Itga3*** **^−/−/^** ***Gpr56*** **^−/−^ brain sections and progenitor cells.**
(TIF)

Figure S2 **Absence of GPR56 protein in mutant mouse brains.**
(TIF)

Figure S3 **GPR56 and α3 integrins are coexpressed in the developing neocortex.**
(TIF)
