# Supplementary figures and images for: GPR56 Functions Together with α3β1 Integrin in Regulating Cerebral Cortical Development
Source: PLoS One. 2013 Jul 9;8(7):e68781. doi: 10.1371/journal.pone.0068781 (PMC3706371; doi:10.1371/journal.pone.0068781)

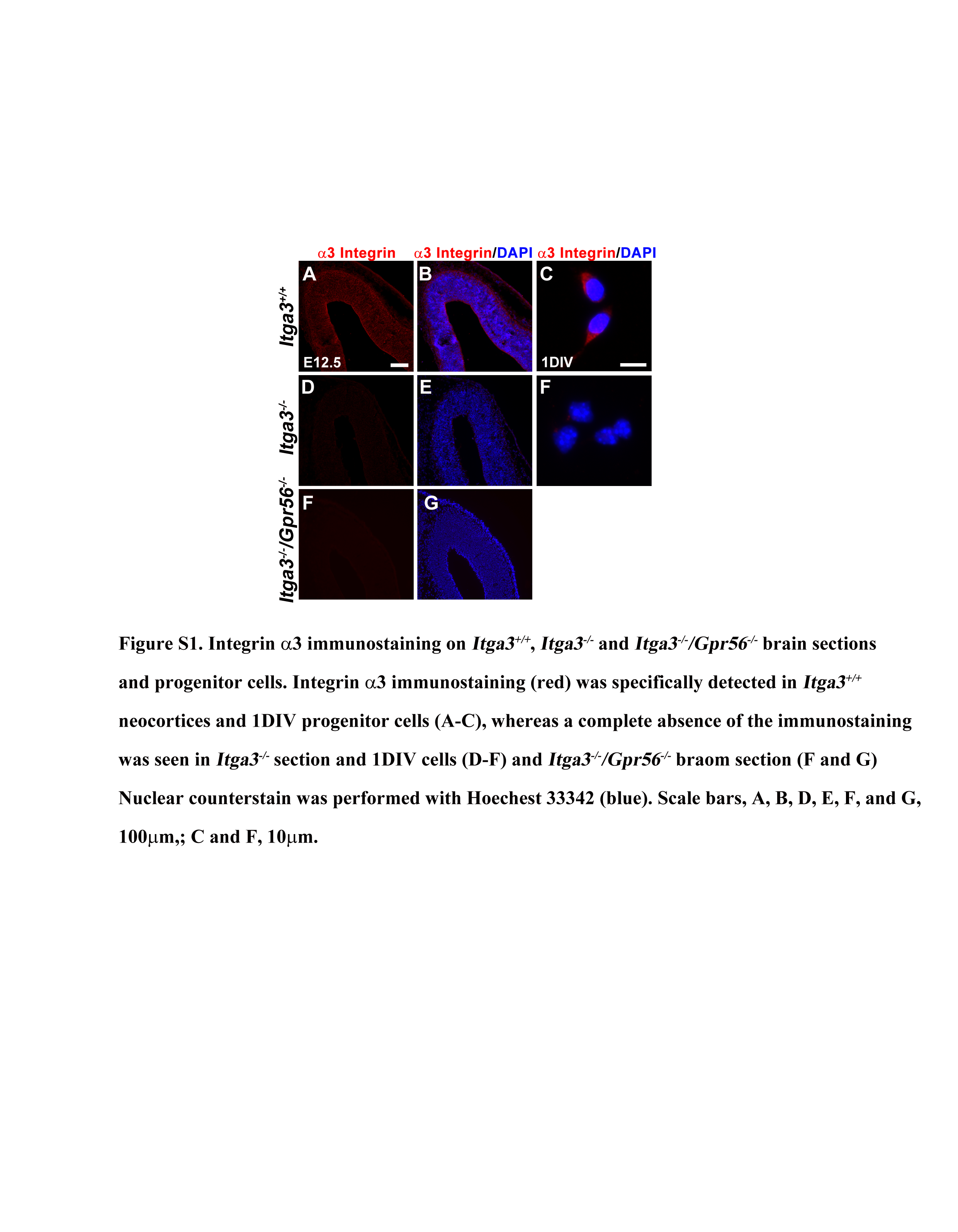

Supplement: Figure S1 — Integrin α3 immunostaining on Itga3 +/+, Itga3 −/−, and Itga3 −/−/ Gpr56 −/− brain sections and progenitor cells. (TIF) [file pone.0068781.s001.tif]

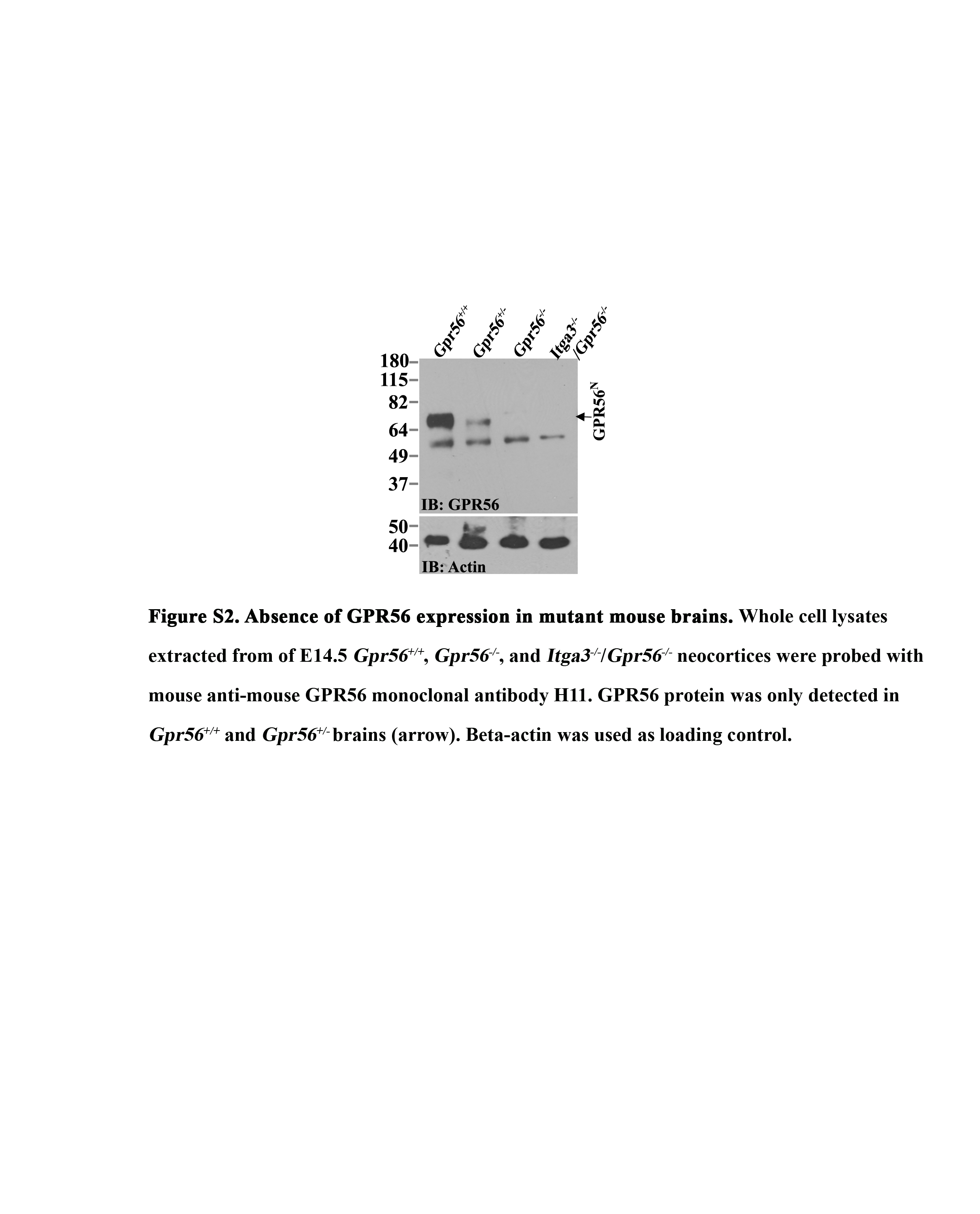

Supplement: Figure S2 — Absence of GPR56 protein in mutant mouse brains. (TIF) [file pone.0068781.s002.tif]

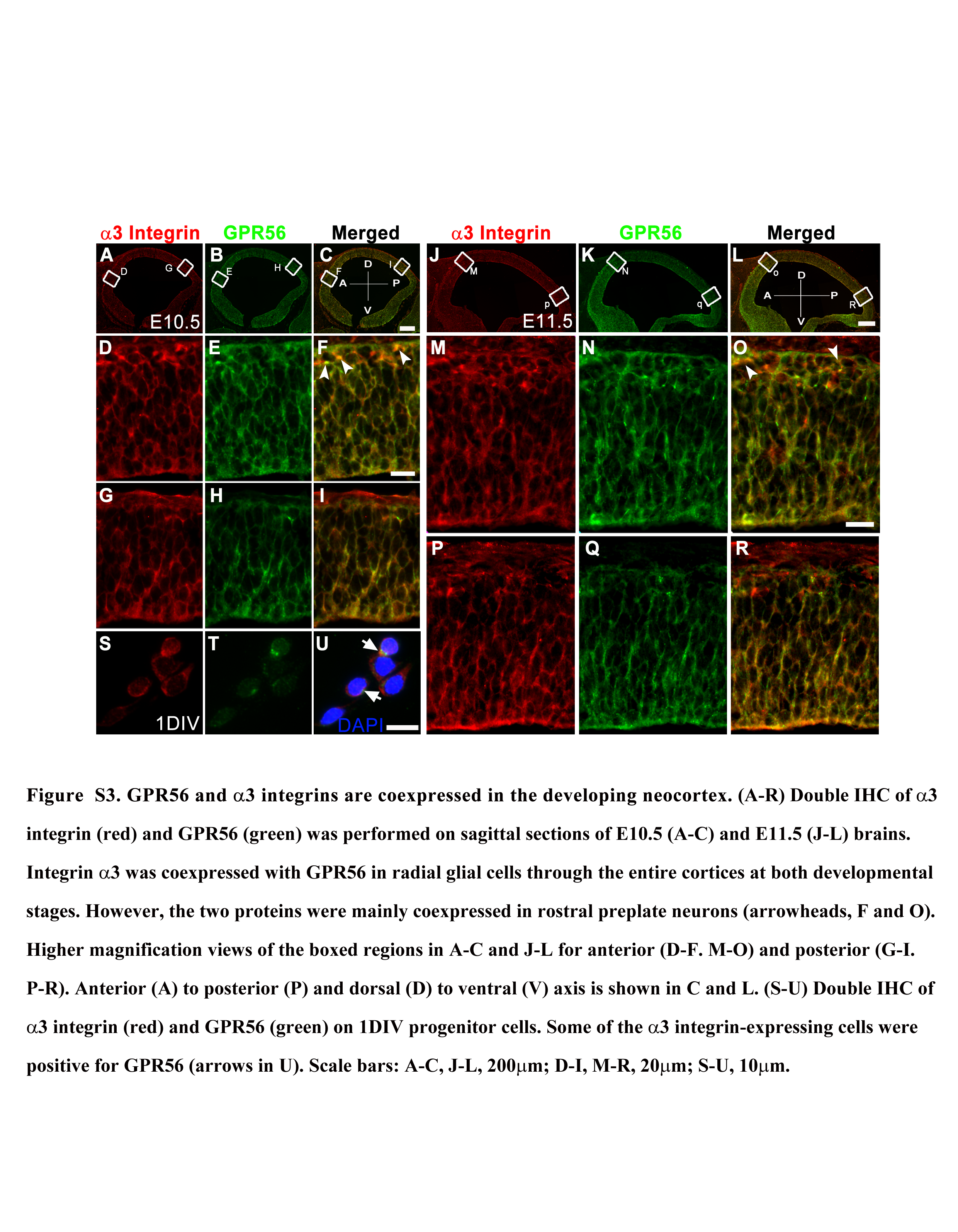

Supplement: Figure S3 — GPR56 and α3 integrins are coexpressed in the developing neocortex. (TIF) [file pone.0068781.s003.tif]
